# Identification of novel 3-dehydroquinate dehydratase (DHQD) inhibitors for anti-tuberculosis activity: insights from virtual screening, molecular docking, and dynamics simulations

**DOI:** 10.1007/s40203-024-00298-x

**Published:** 2025-01-07

**Authors:** Mustafa Alhaji Isa, Abidemi Paul Kappo

**Affiliations:** Molecular Biophysics and Structural Biology (MBSB) Group, Department of Biochemistry, University of Johannesburg, Auckland Park Kingsway Campus, Johannesburg, 2006 South Africa

**Keywords:** DHQD, Docking, MD simulation, ADMET and MM-GBSA

## Abstract

Tuberculosis (TB) remains a pressing global health concern, causing substantial mortality and morbidity despite existing drugs and vaccines. The escalating challenge of drug-resistant TB underscores the critical need for novel medications. This study focuses on the enzyme 3-hydroquinate dehydratase (DHQD) in the shikimate pathway of *Mycobacterium tuberculosis* (Mtb), essential for Mtb growth. Using an in silico approach, the crystal structure of DHQD complexed with 1,3,4-trihydroxy-5-(3-phenoxypropyl)-cyclohexane-1-carboxylic acid (CA) was obtained from the Protein Data Bank. After meticulous preparation, a diverse library of 9699 compounds from Zinc and PubChem databases was subjected to virtual screening, complying with Lipinski's rule of five and compounds capable of binding to DHQD with less binding energy. Molecular docking analysis identified eight compounds with highly favorable binding energies, ranging from -8.99 to -8.39 kcal/mol, surpassing CA's -4.93 kcal/mol. To assess their potential as inhibitors, these eight compounds were subjected to scrutiny for pharmacokinetic properties, encompassing Absorption, Distribution, Metabolism, Excretion, and Toxicity (ADMET). Five compounds (ZINC14981770, ZINC14741224, ZINC14743698, ZINC13165465, and ZINC8442077) demonstrated desirable pharmacokinetic attributes and were selected for further investigation. Subsequent molecular dynamics (MD) simulations and molecular generalized born surface area (MM-GBSA) analyses were conducted. Molecular dynamics (MD) simulations showed that these five compounds formed stable complexes with DHQD over 50 ns, with root mean square deviation (RMSD) values ranging from 1.57 Å to 2.34 Å, indicating high structural stability. In addition, the MM-GBSA binding energy calculations showed that these compounds had favourable binding affinities, with ZINC14981770 exhibiting the lowest free binding energy of -32.70 kcal/mol, followed by ZINC14741224 at -29.67 kcal/mol and ZINC14743698 at -28.79 kcal/mol. These binding energies significantly outperformed the reference compound CA, which had a binding energy of -10.62 kcal/mol. Based on these findings; these five compounds hold promise as potent inhibitors of Mtb DHQD, pending validation through in vitro and in vivo experiments.

## Introduction

Tuberculosis (TB) remains a significant global health concern, causing both high mortality and morbidity rates. Despite the presence of numerous drugs and vaccines, TB continues to claim lives in the 21st century (Isa et al. 2018; Jothieswari and Reddy 2015). The pandemic spread of multi-drug resistant TB (MDR-TB), extensively drug-resistant TB (XDR-TB), and total drug-resistant TB (TDR-TB), along with the co-infection of TB and HIV, presents a serious challenge to TB control efforts (WHO 2023). India, in particular, accounts for more than a quarter of the world's TB cases and deaths (WHO 2023).

Several substantial challenges hinder the effectiveness of TB control programs. These include the cost of drugs, especially second-line anti-TB drugs, which are expensive and require prolonged administration. Besides, a majority of patients treated with second-line drugs often misuse these essential drugs for addressing their drug-resistant TB profiles. Furthermore, many TB drugs are associated with significant side effects. Another critical aspect is that most drugs are ineffective in inhibiting the persistent Mtb population, except for RIF and PZA (WHO 2023). However, a concerning issue is the emergence of Mtb strains with complete resistance for which no available TB drugs can prevent their growth. Strikingly, there have been over ten reports of Total Drug Resistant (TDR) TB cases in India since 2012, where TB is resistant to all existing drugs (Rowland 2012). Hence, the development of new anti-TB drugs capable of inhibiting both actively multiplying bacilli and the non-growing persistent Mtb population is essential to prevent reactivation of the infection.

The shikimate pathway is a metabolic pathway responsible for the synthesis of aromatic amino acids and essential metabolites in various organisms, including bacteria, fungi, algae, plants, and apicomplexan parasites. Its absence in mammals makes it a critical focal point for drug development. This pathway involves seven sequential reactions that ultimately yield chorismate. Chorismate is a key component in Mtb's production of aromatic compounds including mycobactin and ubiquinone as well as amino acids (Zhang et al. 2005). As a result, this route becomes a great target for creating fresh antibacterial drugs to combat Mtb. It is also a desirable target for the creation of novel herbicides and antiparasitic drugs. Any of the enzymes involved in the shikimate pathway can be blocked to stop Mtb growth.

The aroD gene (Herrmann 1995) encodes the enzyme DHQD, which catalyzes the third stage of the shikimate pathway. Through the transformation of 3-dehydroquinate into 3-dehydroshikimate, DHQD aids in a reversible process (Gourley et al. 1999). There are two main varieties of DHQD type I and type II each of which has a different structure and function. By converting quinate into protocatechuate, which serves as a carbon source, these enzymes not only take part in the shikimate route but also play a significant role in the catabolic process.

Mtb utilizes type II DHQD, which catalyzes the biosynthetic pathway through a covalent iminium intermediate to facilitate the dehydration of 3-dehydroquinate via syn elimination (Chaudhuri et al. 1991). Type II DHQD exists as a homododecamer that is heat-stable and is involved in catalyzing both biosynthetic and catabolic pathways. It achieves dehydration via an enolate/enol intermediate and employs a stepwise E1CB mechanism through anti-elimination of water.

In the initial step of the reversible conversion of 3-dehydroquinate into 3-dehydroshikimate, these enzymes remove the pro-S proton from a conserved tyrosine residue, leading to the formation of the enolate intermediate. This intermediate is stabilized by asparagine residues, and a histidine molecule releases a water molecule, resulting in the production of 3-dehydroshikimate (Harris et al. 1993). Inhibiting this enzyme is of paramount importance in controlling the life cycle of MTB. In addition a range of highly effective inhibitors has been documented for this target. Despite the existence of well-established inhibitors, there is a critical need to investigate a broader array of structurally diverse inhibitory compounds. This exploration aims to deepen our understanding of the biological characteristics and uncover potential therapeutic applications (Harris et al. 1993). Hence, this study aims to identify novel inhibitors of DHQD from Mtb through a comprehensive approach involving virtual screening, molecular docking, MD simulation, and MM-GBSA analyses.

## Methodology

### Preparation of DHQD

*Mycobacterium tuberculosis* 3-dehydrogenate dehydratase (DHQD) crystal structure was retrieved from the Protein Data Bank (PDB ID: 3N76), with a resolution of 1.9 Å (Berman et al. 2000). This structure was initially complexed with the ligand 1,3,4-trihydroxy-5-(3-phenoxypropyl)-cyclohexane-1-carboxylic acid (CA). The structure underwent several preparatory steps before molecular docking and simulation studies. First, all bound ligands, cofactors, and non-protein components, such as water molecules, were removed to isolate the protein. Structural refinement was conducted to ensure the integrity of the protein. Missing residues or atoms were identified and inserted, while loops or disordered regions were reconstructed where necessary. Hydrogen atoms were added to ensure proper bonding and stereochemistry, with particular attention given to maintaining the correct chirality of side chains. Next, steric clashes and disulfide bonds were analyzed, with necessary adjustments made to resolve any issues. The refined structure was then subjected to energy minimization and structural optimization to improve the overall geometry and relieve strain. Energy minimization was performed using UCSF Chimera (Pettersen et al. 2004) for visualization and refinement, Swiss-PDB Viewer (Johansson et al. 2012) for side chain and loop adjustments, and Chiron (Ramachandran *et al.,* 2011) for minimizing steric clashes and optimizing bond angles. This refined structure was prepared for subsequent molecular docking, virtual screening, and dynamic simulations.

### Virtual screening (VS)

To identify potential inhibitors of DHQD in *Mycobacterium tuberculosis*, we performed a virtual screening (VS) process using a curated library of 9,699 compounds from the Zinc and PubChem databases. The rationale behind selecting these compounds is their diverse chemical structures and representation in publicly available databases widely used in drug discovery research. These databases provide compounds with basic drug-like properties, making them suitable for early-stage drug discovery screening. Virtual screening was performed using the RASPD^+^ tool (Holderbach et al. 2020), a computationally efficient protocol that identifies lead-like molecules by predicting binding affinity between the compounds and the target protein (He et al. 2012), which in this case was DHQH. The 3D structure of DHQH, along with its defined ligand-binding pocket, was used as the basis for this analysis. A total of 9,699 compounds from the PubChem and Zinc databases showed that minimum binding energy was obtained. Following this, we employed PyRx 8.0 to confirm the binding energies of the compounds further. PyRx was chosen due to its efficiency in molecular docking and energy calculation, ensuring the identification of ligands with the minimum binding energies. This step allowed us to prioritize compounds with the highest likelihood of favourable binding to DHQD.

Beyond Lipinski's rule, the compounds were subjected to additional filtering using the DataWarrior tool (Sander et al. 2015). This stage was critical as it incorporated various physicochemical characteristics, including molecular weight, lipophilicity (logP), hydrogen bond donors (HBD), and hydrogen bond acceptors (HBA). This meticulous refinement aimed to pinpoint compounds exhibiting the desired attributes. Subsequently, the selected compounds were employed in the molecular docking process using Autodock 4.0 (Morris et al. 1998).

### Molecular docking analysis

The molecular docking studies used Autodock 4.2 (Morris et al. 1998), widely recognized software for predicting protein-ligand interactions. This study investigated the binding orientation and affinity of various ligands to 3-Dehydroquinate Dehydratase (DHQD) from *Mycobacterium tuberculosis*. The ligands were selected based on their favourable physicochemical properties, including molecular weight, hydrogen bond donor (HBD) and acceptor (HBA) values, and adherence to Lipinski’s rule of five, ensuring drug-like characteristics. The selection process was further refined using the DataWarrior tool (Sander et al. 2015), which filtered out ligands with unfavourable pharmacokinetic or toxicity profiles. To validate the docking protocol and establish a control for comparison, DHQD was also docked with its known inhibitor, carboxylic acid (CA), which had been co-crystallized with the protein in previous experimental studies. This allowed the docking accuracy to be benchmarked, with the binding energy and orientation of CA serving as a reference for new ligands.

The molecular docking simulations employed the Lamarckian Genetic Algorithm (LGA), a method known for effectively predicting ligand conformations by combining local minimization with genetic crossover and mutation techniques. This approach was chosen for its ability to accommodate ligand flexibility, ensuring accurate binding orientation estimations and interaction-free energy. The DHQD receptor was prepared by assigning Kollman charges and adding polar hydrogens to optimize ligand interactions. Ligand files were converted to PDBQT format, which encodes atomic types, partial charges, and torsional degrees of freedom. While the protein was kept rigid to maintain structural integrity, ligands were allowed complete torsional flexibility to explore possible conformations within the binding site.

A grid box was generated to define DHQD’s active site, with dimensions of 60 × 60 × 60 Å and a grid spacing of 0.375 Å, ensuring that the entire binding pocket and surrounding regions were covered for potential ligand binding. The structural analysis showed that eight critical residues, Glu20, Gly25, Ala76, Thr82, Ala89, Ile102, Val105, and Arg112, make up the active and substrate-binding site of the enzyme. These key residues were included in the grid box to make sure the docking simulations focused on the right area for potential ligand interactions. A total of 10 independent docking runs were performed for each ligand, with a population size of 150 individuals in the genetic algorithm. The docking simulations were set to run for 27,000 generations and 2,500,000 evaluations, comprehensively exploring the conformational space. These parameters were optimized to balance computational efficiency and accuracy (Kumari and Dalal 2022).

For each docking run, the final binding conformation was determined based on the lowest binding free energy (ΔG_bind_), which represents the interaction strength between the ligand and the protein. The binding free energy was calculated as the sum of several components: van der Waals energy (ΔG_vdw_), representing dispersion interactions between non-polar regions; electrostatic energy (ΔG_elect_), reflecting Coulombic interactions between charged or polar regions; hydrogen bond and desolvation energy (ΔG_hbond_), capturing the energy costs of desolvating the ligand and protein and forming hydrogen bonds; conformational internal energy (ΔG_conform_), representing energy changes due to ligand conformational adjustments upon binding; torsional free energy (ΔG_tor_), accounting for the ligand’s rotational freedom around specific bonds; and unbound system energy (ΔG_solv_), calculating the solvation energy of the unbound ligand in the solvent.

Post-docking analysis was conducted by calculating the Root Mean Square Deviation (RMSD) values for the docked poses, assessing the accuracy and consistency of the docking results. An RMSD threshold of 2.0 Å was used to determine the similarity of docked conformations to the reference structure, which ensure that the docking algorithm consistently identified accurate binding modes. Additionally, the binding energies of the newly docked ligands were compared to that of the control ligand (CA) to identify any compounds with superior binding affinity or distinct interaction patterns that could improve their inhibitory potential. The docked poses were visually inspected using PyMOL to analyze critical interactions that contribute to ligand binding stability, such as hydrogen bonds, π-π stacking, and hydrophobic contacts.

### Pharmacokinetics analysis

In drug discovery and development, many compounds fail to progress to clinical trials due to unfavourable pharmacokinetic properties, including issues related to absorption, distribution, metabolism, excretion (ADME), and toxicity (Isa et al. 2018). Therefore, conducting broad in silico ADME and toxicity profiling at the early stages of the drug development process is essential. This study employed a combination of predictive computational tools, including AdmetSAR (Cheng et al. 2012), DataWarrior (Sander et al. 2015), and ADME/TOX programs (Lipinski et al. 1997; Veber et al. 2002), to assess the pharmacokinetic properties of the selected ligands. These tools were chosen based on their accuracy in predicting crucial pharmacokinetic parameters, thereby helping to optimize compound selection and minimize the risk of failure in clinical trials. The AdmetSAR tool was employed to predict various ADME properties, including human intestinal absorption (HIA), blood–brain barrier (BBB) permeability, plasma protein binding (PPB), and cytochrome P450 (CYP450) inhibition profiles. These predictions are critical for determining a compound's ability to be absorbed into the systemic circulation, distributed to target tissues, and metabolized without interference with major enzymes such as CYP450, which plays a crucial role in drug metabolism. Specifically, the potential inhibition of the CYP450 2D6 isoform was evaluated, as this enzyme is responsible for the metabolism of many drugs and is a known source of drug-drug interactions.

The ADME/TOX program was used to predict toxicity-related parameters such as AMES toxicity (a widely accepted test for mutagenicity), hepatotoxicity, tumorigenicity, skin irritation, and reproductive toxicity. These predictions are critical for anticipating adverse effects during clinical testing. The AMES test, in particular, evaluates the mutagenic potential of compounds, helping to rule out those that could pose a carcinogenic risk.

### Molecular dynamic (MD) simulation analysis

To investigate the dynamic behaviour of DHQD bound to its ligands, including the previously co-crystallized ligand carboxylic acid (CA), molecular dynamics (MD) simulations were performed using the AMBERTOOLS18 (Case et al. 2023). MD simulations provide a detailed understanding of the stability, flexibility, and conformational changes of the protein-ligand complexes over time, which are critical for understanding their binding interactions and potential drug-like properties. The 3D structures of the DHQD-ligand complexes were first prepared by adding explicit hydrogen atoms, ensuring that the protonation states were appropriate for the physiological pH. The pKa values of ionizable residues were calculated using the PROPKA server to ensure accurate protonation at physiological conditions. The *Antechamber* module of AMBER was employed to generate missing ligand parameters necessary for the subsequent MD simulations. Atomic charges for the ligands were calculated using the RESP fitting procedure at the HF/6-31G(d) level of theory, ensuring high accuracy for molecular interactions during simulations. The leap program was employed for the protein and ligand systems to create the required coordinate and topology files. Force fields were assigned to the protein using the General AMBER Force Field (GAFF) and the ff12SB force field for the ligands. GAFF and ff14SB were selected based on their proven reliability in modelling proteins and small organic molecules in complex biological environments. The entire protein-ligand complex was solvated in a TIP3P water model within an octahedral box to simulate the system in a biologically relevant aqueous environment. The box was large enough to ensure a minimum of 10 Å of water between the solute and the edge of the simulation box. Sodium ions were added to neutralize the system, ensuring that electrostatic artefacts were minimized during the simulation. Periodic boundary conditions were applied to maintain system integrity during the simulation.

The system underwent a two-step minimization process to remove unfavourable steric clashes and prepare for the production MD run. In the first step, positional restraints of 544 kcal/mol were applied to the protein-ligand complex, and a 5000-step minimization was performed, alternating between the steepest descent and conjugate gradient methods (2500 steps each). This was done to relax the solvent while keeping the complex rigid. In the second step, these positional restraints were removed, and the entire system underwent another 5000-step minimization (2500 steps of steepest descent followed by 2500 steps of conjugate gradient), allowing for the relaxation of both the protein and the ligands. Convergence criteria of 0.01 kcal/mol/Å were used to ensure sufficient minimization.

Following energy minimization, the system was gradually heated from 0 K to 300 K using the Langevin dynamics temperature regulation method. This heating process was carried out over 100,000 steps with a collision frequency of 1 ps⁻^1^. The Langevin thermostat was chosen to ensure proper temperature control while avoiding artefacts that could arise from other thermostats. The heating phase was conducted without applying pressure control to stabilize the system at the desired temperature. Once the system reached 300 K, it was equilibrated at a constant temperature of 300 K and constant pressure of 1 atm. The Berendsen barostat was applied during this phase to regulate the pressure. A time step of 2 fs was employed for all MD simulations to maintain accuracy while ensuring computational efficiency. The Particle Mesh Ewald (PME) method was used to handle long-range electrostatic interactions. The SHAKE algorithm was used to constrain all bonds involving hydrogen atoms, which allowed for a more significant time step and reduced computational cost without sacrificing accuracy.

The production MD simulations were conducted for a total duration of 50 ns, providing sufficient time to assess the dynamic behaviour of the DHQD-ligand complexes and their stability. Throughout the simulation, the protein–ligand complex's root mean square deviation (RMSD) was examined to evaluate its stability. The RMSD is a standard parameter for assessing the structural stability of the protein–ligand complex over time. A stable RMSD profile suggests the complex remains within a consistent conformation during the simulation, indicating that the ligand is stably bound within the active site. Moreover, each residue's root mean square fluctuation (RMSF) was calculated to assess the flexibility of specific regions within the protein, particularly those involved in ligand binding. RMSF provides an understanding of which amino acid residues deviate from their mean positions during the simulation, which can highlight flexible regions or regions critical for binding stability. RMSF analysis was performed using the CPPtraj module of the AMBERTOOLS18 package, providing detailed residue-level understandings of the dynamic movements within the protein–ligand complex.

To assess the statistical significance of the simulation results over the entire 50 ns period, a block analysis was performed using the CPPTRAJ module. The simulation data for RMSD, RMSF, and Rg were divided into blocks of 5 ns, and statistical comparisons were made to determine whether the observed differences were statistically significant. The standard error of the mean (SEM) was calculated for each block to ensure robust comparisons of structural stability and flexibility metrics.

### Free binding energy (MM-GBSA) analysis

A crucial technique for quantifying the interactions between proteins and ligands is the MM-GBSA (Genheden et al. 2015). The technique, which has had great success over the years (Ghasemi et al. 2016), uses the MD simulation approach to ascertain the free binding energy of the protein-ligand complex. MM-GBSA offers the advantage of balancing computational efficiency with accuracy, making it ideal for ranking ligand binding affinities in drug discovery projects. In this study, the free binding energy of the DHQD-ligand complexes was calculated using Amber14 for the MM-GBSA analysis based on an average of 500 snapshots taken at intervals of 10 ps from the trajectory of the last 5 ns of the molecular dynamics simulation. Snapshots were extracted exclusively from the equilibrated portion of the trajectory, ensuring that only stable conformations of the protein-ligand complex were analyzed. After confirming system equilibration, the final 5 ns segment was selected by monitoring root mean square deviation (RMSD) and energy convergence**.** The procedure is condensed in the equation below.

1 $$ \Delta G_{binding} = {\text{G}}_{complex} - \left( {{\text{G}}_{receptor} + {\text{G}}_{ligand} } \right) $$

2 $$ G_{x} = E_{MM} + G_{solv} - T\Delta S $$

3 $$ E_{MM} = E_{vdW} + E_{ele} $$

4 $$ G_{solv} = G_{polar} + G_{nonpolar} $$

5 $$ \Delta G_{{{\text{MM}} - {\text{GBSA}}}} = \Delta G_{{{\text{vdw}}}} + \Delta G_{{{\text{elec}}}} + \Delta G_{{{\text{polar}}}} + \Delta G_{{{\text{nonpolar}}}} $$

The total binding energy was determined from each snapshot based on the difference between the free binding energy of the complex (*G*_*complex*_), receptor (*G*_*receptor*_) and the ligand (*Gl*_*igand*_), as shown in Eq. (1). The free binding of each component (*G*_*x*_) was calculated using the sum of configurational entropy *T*$\Delta$*S,* the sum of solvation binding energy (*G*_*sol*_), and the sum of molecular mechanical gas-phase free binding energy (*E*_*MM*_) (Eq. 2). The sum of the molecular mechanical gas-phase free binding energy (*E*_*MM*_) further gave rise to the van der Waals energy (*E*_*vdW*_) and Gas-phase electrostatic energy (*E*_*ele*_) (Eq. 4). Lastly, total free binding energy was calculated based on the values of the gas-phase electrostatic energy (*E*_*ele*_), van der Waals (*E*_*vdW*_) polar (*G*_*polar*_) and nonpolar (*G*_*nonpolar*_) component (equation (Eq. 5).

## Results and discussion

### Virtual screening and molecular docking of DHQD

**Fig. 1:**
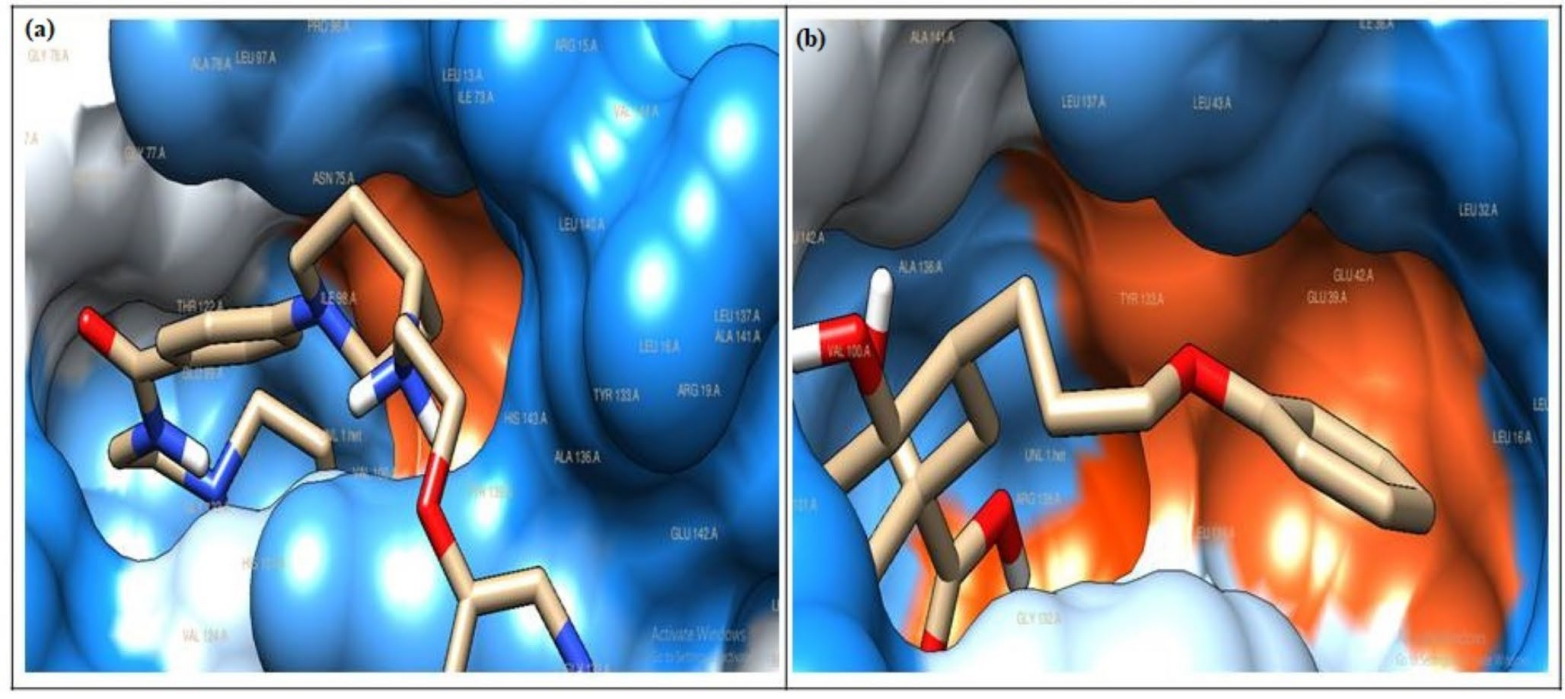
3D interactions of the ligand with the least binding energy and the reference ligand **a** ZINC14981770 **b** CA

**Fig. 2:**
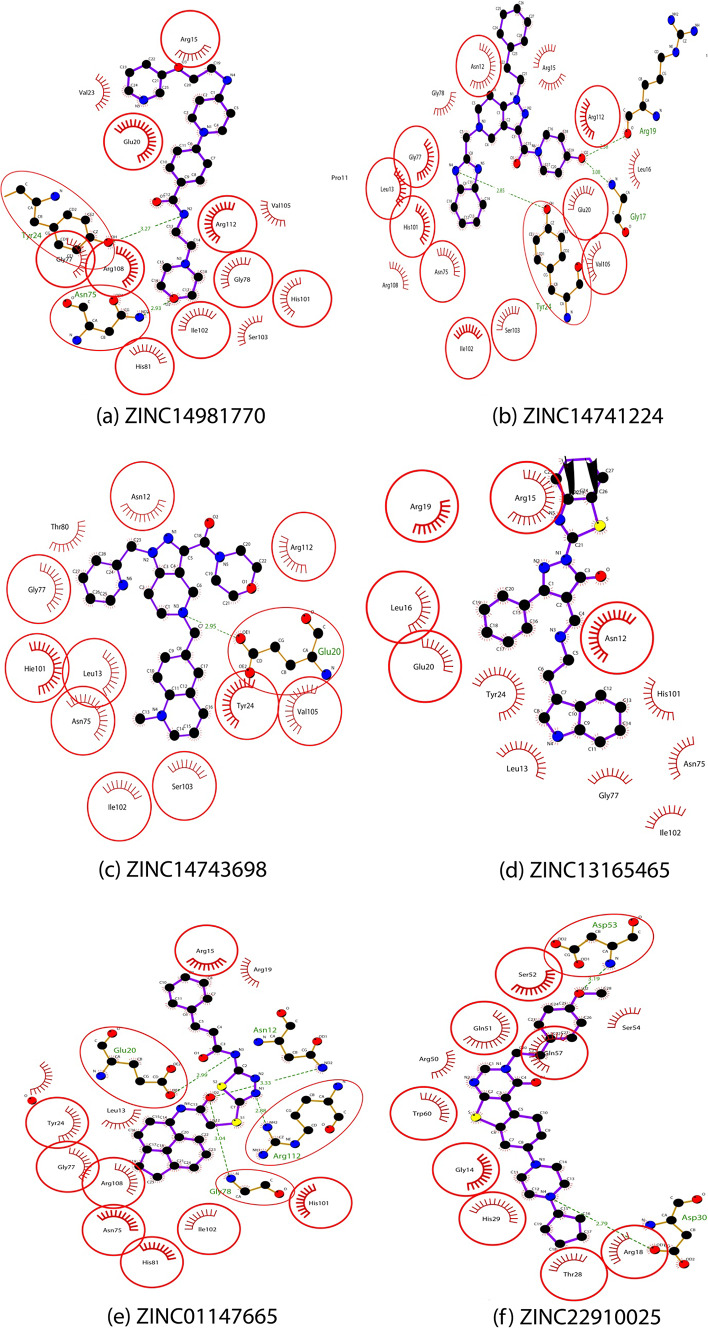

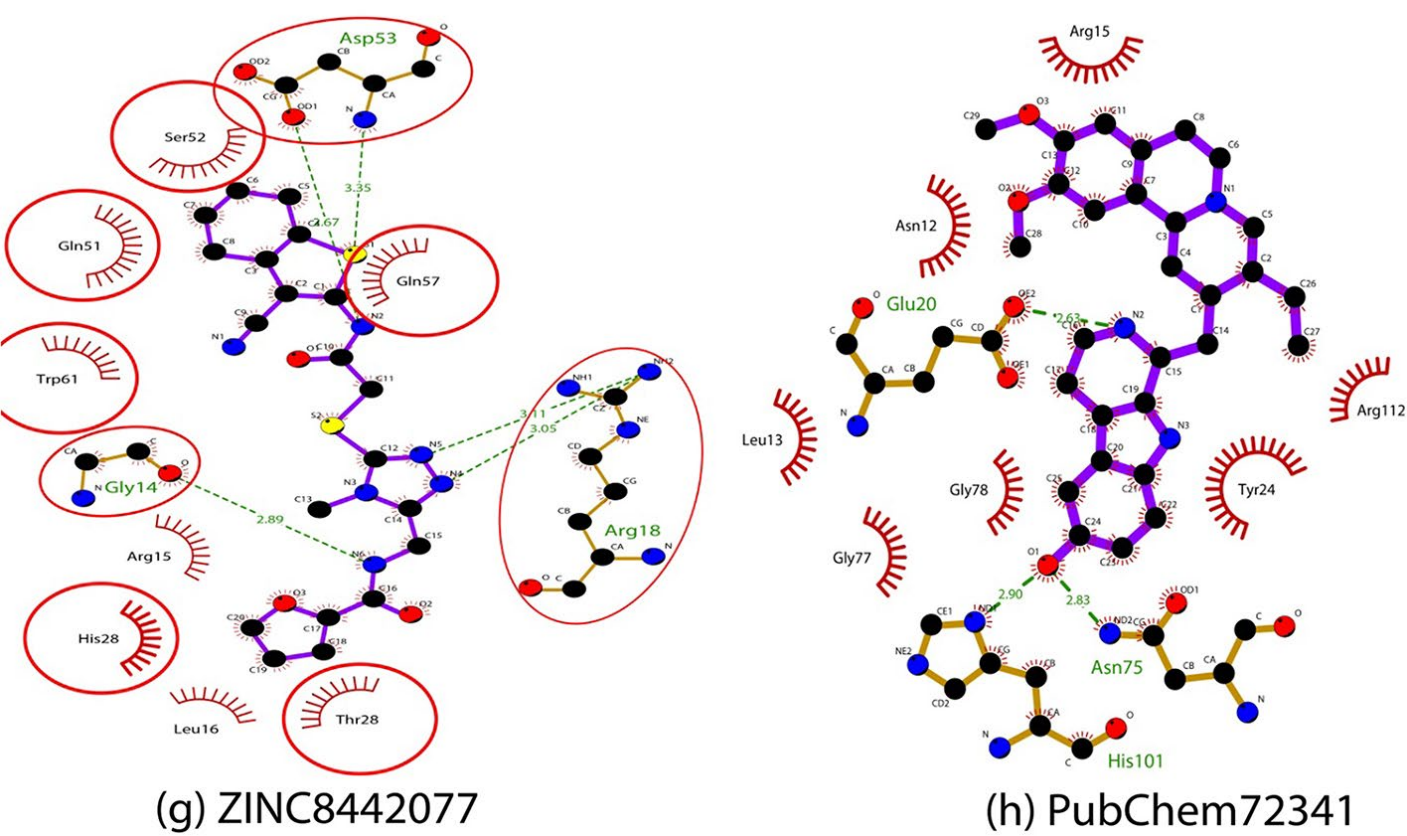
The illustration of the hydrogen and hydrophobic interactions between the chosen ligands and DHQD

The virtual screening and molecular docking studies conducted in this research aimed to identify potential inhibitors for DHQD, a vital enzyme in Mtb (MTB), by analyzing 9,699 compounds from the PubChem and Zinc databases. After an initial screening process based on Lipinski's rule of five, compounds were docked into the active site of DHQD, where they were evaluated for their binding affinities and interatomic interactions (Table 1). Also, the docked poses of these compounds were validated by calculating the RMSD relative to the crystal pose of CA, which focus on the heavy atoms. All RMSD values were below the 2.0 Å threshold, which confirm the reliability of the docking protocol and structural alignment with the active site. The docking scores, ranging from -8.99 to -8.39 kcal/mol, revealed that eight compounds exhibited better binding energies than the reference ligand carboxylic acid (CA), which had a binding energy of -4.93 kcal/mol (Table 2). This suggests that these compounds have more substantial potential to inhibit DHQD, thus disrupting the Mtb metabolic pathway. This is particularly significant given the role of DHQD in the shikimate pathway, which is essential for synthesising secondary metabolites critical for Mtb survival and pathogenesis**.** With the minimum binding energy of -8.99 kcal/mol, ZINC14981770 demonstrated a strong interaction profile with DHQD. The two critical hydrogen bonds were formed between the ligand and the hydroxyl group of Tyr24 (distance = 3.27 Å) and the polar amide of Asn75 (distance = 2.96 Å) (Table 2) (Fig. 1a). These residues are crucial for DHQD’s catalytic mechanism, which involves substrate binding and electron transfer during the reaction. The ligand's RMSD was 1.85 Å, indicating excellent alignment with the crystal pose. The formation of these hydrogen bonds suggests a stable interaction with the enzyme's active site, and this stability could disrupt the enzyme's normal function, preventing the proper positioning of the substrate and halting the catalysis process. Besides, the hydrophobic interactions with residues such as Arg15, Glu20, Gly78, His81, Ser103, and Val105 contribute to the overall binding affinity, providing a complementary fit that stabilizes the ligand within the active site (Fig. 2a). These hydrophobic contacts are essential not only for reducing the entropic cost of ligand binding but also for ensuring the ligand’s prolonged residence in the active site, which is key to its inhibitory effect. The combination of these hydrogen bonds and hydrophobic interactions likely inhibits the catalytic activity of DHQD, reducing its ability to facilitate downstream Mtb metabolic processes. As DHQD is integral to Mtb's biosynthesis of essential compounds, inhibiting this enzyme could effectively reduce Mtb's growth and virulence. Similarly, ZINC14741224 had a binding energy of -8.98 kcal/mol, and exhibited a network of three hydrogen bonds, which include interactions with Arg19 (2.38 Å), Gly17 (3.08 Å), and Tyr24 (2.85 Å) (Table 2). The hydrogen bond with Arg19 is essential as this residue lies close to the substrate binding site, suggesting that the ligand could effectively block substrate access. Hydrophobic interactions with residues such as Asn12, Arg15, His101, and Gly78 further contribute to the ligand's stabilization within the binding pocket (Fig. 2b). With an RMSD of 1.92 Å, ZINC14741224 appears to block the enzyme, thereby inhibiting its function, preventing substrate processing and thus inhibiting Mtb growth. Also, ZINC01147665 had a binding energy of -8.57 kcal/mol and formed four hydrogen bonds with Asn12 (3.33 Å), Glu20 (2.99 Å), Gly78 (3.04 Å), and Arg112 (2.88 Å) (Table 2). These interactions occur at critical catalytic residues, indicating that ZINC01147665 could disrupt the proper positioning of essential substrates within the active site. Hydrophobic interactions with Arg15, Tyr24, and His81 significantly add to the overall stabilization of the ligand (Fig. 2e). Also with RMSD of 1.89 Å validated its structural alignment. Multiple hydrogen bonds and hydrophobic contacts suggest that ZINC01147665 could be a potent inhibitor of DHQD, interfering with the enzyme's critical role in the shikimate pathway, essential for synthesizing secondary metabolites in Mtb.

**Table 1:** Characteristics and Drug-like Properties of the Chosen Ligands

S/No.	Compound ID	Compound Name	Molecular weight (≤ 500)	No. of HBA(≤ 10)	No. of HBD (≤ 5)	LogP (< 5)
1	ZINC14981770	N-(2-morpholinoethyl)-4-[4-[2-(3-pyridyloxy)ethylamino]-1-piperidyl]benzamide	453.3	6	2	1.9
2	ZINC14741224	[5-(1H-benzimidazol-2-ylmethyl)-1-phenethyl-6,7-dihydro-4H-pyrazolo[4,5-c]pyridin-3-yl]-(4-hydroxy-1	484.6	8	2	2.1
3	ZINC14743698	[5-[(1-methyl-3,4-dihydro-2H-quinolin-6-yl)methyl]-1-(2-pyridylmethyl)-6,7-dihydro-4H-pyrazolo[4,5-c	487.6	8	1	-0.7
4	ZINC13165465	(4R)-2-(1,3-benzothiazol-2-yl)-4-[(E)-2-(1H-indol-3-yl)ethyliminomethyl]-5-phenyl-4H-pyrazol-3-one	464.6	6	3	3.8
5	ZINC01147665	(E)-N-[5-[[2-(acenaphthen-5-ylamino)-2-keto-ethyl]thio]-1,3,4-thiadiazol-2-yl]-3-phenyl-acrylamide	472.1	6	2	5.7
6	ZINC22910025	(7S)-7-(4-cyclopentylpiperazin-1-yl)-3-[2-(4-methoxyphenyl)ethyl]-5,6,7,8-tetrahydrobenzothiopheno[3	492.3	6	0	5.4
7	ZINC8442077	N-{[5-({2-[(3-cyano-4,5,6,7-tetrahydro-1-benzothien-2-yl)amino]-2-oxoethyl}sulfanyl)-4-methyl-4H-1,2,4-triazol-3-yl]methyl}-2-furamide	456.1	8	2	2.0
8	PubChem72341	Tubulosine	475.3	5	3	4.1

**Table 2:** Results from the docking of DHQD with ligands with good binding energies

S/No	Zinc code	Energy of binding (kcal/mol)	Interacting residues	Hydrogen bond distance (Å)	RMSD from crystal pose (Å)
1	ZINC14981770	─8.99	Tyr24 Asn75	3.27 2.96	1.85
2	ZINC14741224	─8.98	Gly17 Arg19 Tyr24	3.08 2.38 2.85	1.92
3	ZINC14743698	─8.87	Glu20	2.95	1.99
4	ZINC13165465	─8.77	0	0	2.10
5	ZINC01147665	─8.57	Glu20 Gly78 Arg112 Asn12	2.99 3.04 2.88 3.33	1.89
6	ZINC22910025	─8.48	Asp53 Asp30	3.19 2.79	2.05
7	ZINC8442077	─8.41	Asp53 Asp53 Arg18 Arg18 Gly14	3.35 2.67 3.05 3.11 2.89	1.96
8	PubChem72341	─8.39	His101 Asn75 Glu20	2.90 2.83 2.63	1.88
9	CA	─4.93	His81 Asn75 His101 Arg112	3.15 2.78 2.96 3.09	-

ZINC22910025, with a binding energy of -8.48 kcal/mol, ZINC22910025 primarily interacts with DHQD via two hydrogen bonds with Asp53 (3.19 Å) and Asp30 (2.79 Å). These acidic residues are strategically located near the active site, and their interaction with the ligand could alter the protonation state and overall activity of the enzyme. An RMSD of 2.05 Å, which I slightly higher, remains within acceptable limits, confirming its docking accuracy. Although ZINC22910025 exhibits fewer hydrogen bonds than other ligands, the strong hydrogen bonds with these catalytic residues suggest it could still effectively inhibit DHQD activity. The hydrophobic interactions with surrounding residues further improve its stability in the binding pocket. Also, ZINC8442077 demonstrated a binding energy of -8.41 kcal/mol and formed five hydrogen bonds, including interactions with Asp53 (3.35 Å and 2.67 Å), Gly14 (2.89 Å), and Arg18 (3.05 Å and 3.11 Å). The dense hydrogen bond network indicates a strong affinity for the active site, potentially impeding substrate entry or product release. The hydrophobic interactions with Arg15 and Leu16 further stabilize the ligand (Fig. 2g). The combination of hydrogen bonding and hydrophobic interactions suggests that ZINC8442077 could effectively inhibit DHQD by blocking critical catalytic residues involved in the enzyme's function. Finally, PubChem72341 had a binding energy of -8.39 kcal/mol, and PubChem72341 formed three hydrogen bonds with His101 (2.90 Å), Glu20 (2.63 Å), and Asn75 (2.83 Å). These hydrogen bonds are crucial for stabilizing the ligand at the active site, potentially preventing the enzyme from achieving the conformational changes required for catalysis. Hydrophobic interactions with residues such as Arg15, Tyr24, and Gly77 increase the ligand's binding stability (Fig. 2h). The position of PubChem72341 within the binding pocket suggests it could interfere with the catalytic mechanism of DHQD, limiting its role in Mtb's biosynthetic pathways.

The results of this study indicate that the identified compounds possess strong binding affinities to DHQD, better than the binding energy of the reference ligand CA (Fig. 1b). The detailed interaction profiles, including hydrogen bonding, electrostatic interactions, and hydrophobic contacts, suggest that these ligands could serve as effective inhibitors of DHQD, thereby disrupting the biosynthetic pathways crucial for Mtb survival. By targeting residues in the active site essential for catalysis, these ligands could hinder the production of mycobacteria and other critical secondary metabolites required for Mtb pathogenesis.

The strong interactions observed, particularly with residues like Arg15, Tyr24, Glu20, and Asn75, which play pivotal roles in substrate binding and enzyme function, indicate that these ligands could effectively block the enzyme's activity. Furthermore, the identification of these critical binding interactions opens avenues for designing compounds that could overcome potential resistance mechanisms, such as mutations in the active site or efflux pump activation, that often limit the efficacy of current TB drugs. The hydrophobic interactions further stabilize the ligands within the active site, making them potential candidates for drug development. The findings suggest that these compounds could be developed into potent anti-TB drugs.

The results of the docking study highlight the promising potential of several compounds as inhibitors of DHQD, a key enzyme in the metabolic pathway of Mtb. Through virtual screening and molecular docking of 9,699 compounds, we identified several candidates that exhibited superior binding affinities compared to the reference ligand carboxylic acid, with the top compound, ZINC14981770, showing a binding energy of -8.99 kcal/mol. The analysis revealed that the identified inhibitors, including ZINC14981770 and ZINC14741224, engage in multiple critical interactions, such as hydrogen bonds and hydrophobic contacts, with essential residues in the enzyme's active site. These interactions not only stabilize the ligands within the binding pocket but also suggest mechanisms through which they could hinder DHQD’s catalytic function, thereby disrupting Mtb's metabolic processes. The formation of hydrogen bonds with catalytic residues, as seen with compounds like ZINC01147665 and ZINC22910025, indicates that these ligands could effectively block substrate access or alter the enzyme's protonation state, further impeding its activity.

### ADMET analysis of selected ligands interacted with DHQD

One vital aspect of the metabolic profile is the inhibition of CYP450 enzymes, particularly CYP450 2D6, which is involved in the metabolism of many drugs. Inhibition of this enzyme can lead to drug-drug interactions and adverse effects. Among the eight compounds, ZINC22910025 was identified as a CYP450 2D6 inhibitor, which is undesirable. Inhibitors of this enzyme are often excluded in drug development due to the risk of accumulating toxic metabolites or interactions with other drugs metabolized by the same pathway. Given that ZINC22910025 exceeded the acceptable threshold for CYP450 inhibition (AC50 > 57 μM), this compound was excluded from further analysis.

Toxicity tests, including the Ames test, carcinogenicity, and mutagenicity predictions, revealed that most compounds demonstrated good safety profiles. However, PubChem72341 and ZINC01147665 exhibited high mutagenic, tumorigenic, and irritant properties, indicating potential health risks. The presence of such toxicity markers is a red flag in drug development, as compounds exhibiting mutagenic or carcinogenic potential could contribute to long-term adverse effects, such as cancer. Consequently, these two compounds were excluded from further studies.

Eliminating three compounds (ZINC01147665, ZINC22910025, and PubChem72341) based on unfavourable ADMET properties emphasizes integrating pharmacokinetics into early drug discovery stages. Despite these compounds showing strong binding affinities in molecular docking, their ADMET profiles revealed significant limitations that would likely fail in preclinical or clinical trials.

The remaining five compounds (ZINC14981770, ZINC14741224, ZINC14743698, ZINC13165465, and ZINC8442077) met all the necessary ADME and toxicity criteria, making them suitable candidates for further analysis, including molecular dynamics (MD) simulations. These compounds demonstrated strong binding affinities and presented favourable pharmacokinetic profiles, making them promising leads for future drug development targeting DHQD.

The physicochemical properties of these compounds, such as logP (lipophilicity), molecular weight, and hydrogen bonding capacity, directly influenced their absorption and distribution. The strong HIA and BBB penetration properties observed for all compounds can be attributed to their optimal balance of lipophilicity and molecular size, characteristics critical for oral bioavailability and crossing biological membranes (Pires et al. 2015).

Furthermore, these physicochemical properties also correlate with the binding affinities observed in the molecular docking studies. Hydrophobic interactions, hydrogen bonding, and van der Waals forces between the ligands and DHQD's active site are likely influenced by these properties. ZINC14981770, which had the highest binding affinity (-8.99 kcal/mol), exhibited significant interactions with critical residues at the DHQD binding site, including Tyr24, Asn75, and Ser103, forming both hydrophobic and hydrogen bonds.

The molecular docking and ADMET analysis provided a valuable understanding of the suitability of these compounds as potential DHQD inhibitors. While three compounds were eliminated due to unfavourable pharmacokinetic or toxicity properties, the remaining five compounds demonstrated therapeutic solid potential and are considered for the MD simulation.

### Molecular dynamic simulation analysis

Following the outcomes of the ADME and toxicity analyses, five ligands (ZINC14981770, ZINC14741224, ZINC14743698, ZINC13165465, and ZINC8442077) were selected for molecular dynamics (MD) simulations based on their favourable pharmacokinetic profiles. These compounds exhibited binding energies ranging from ─8.99 to ─8.41 kcal/mol, which suggested promising binding affinities. Besides, the reference ligand, CA, previously co-crystallized with DHQD (Dehydroquinase), was included in the MD simulations for comparative analysis. The simulation evaluated these ligand–protein complexes' stability, conformational changes, and interaction dynamics over a 50 ns period (Suvaithenamudhan, and Parthasarathy 2017, 2023; Kumari et al. 2022).

To assess the stability of the six complexes (DHQD─ZINC14981770, DHQD─ZINC14741224, DHQD─ZINC14743698, DHQD─ZINC13165465, DHQD─ZINC8442077, and DHQD─CA), Root Mean Square Deviation (RMSD) was employed as a primary metric. RMSD measures the deviation of atomic positions from their initial coordinates, providing an understanding of the overall structural stability and conformational changes. In addition to RMSD, Root Mean Square Fluctuation (RMSF) was used to analyze the flexibility of specific amino acid residues. At the same time, the radius of gyration (Rg) was calculated to evaluate the compactness of the complexes, indicating whether the protein–ligand systems remained folded or unfolded during the simulation.

**Fig. 3:**
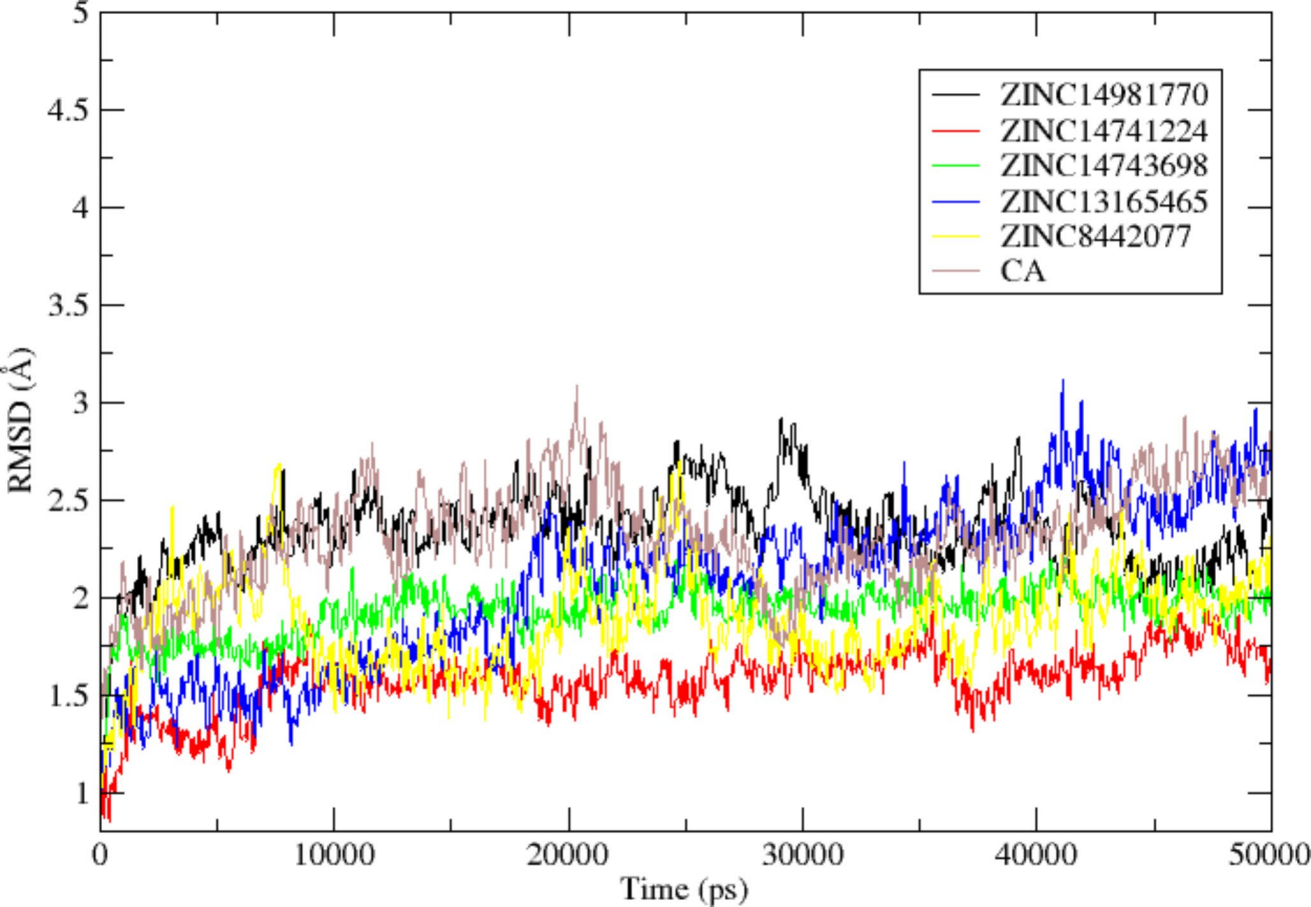
displays the 50 ns MD simulation results (RMSD analysis) for the DHQD─ZINC14981770, DHQD─ZINC14741224, DHQD─ZINC14743698, DHQD─ZINC13165465, DHQD─ZINC8442077, and DHQD─CA complexes

The RMSD values of the five selected ligand complexes and the reference DHQD─CA complex were analyzed to evaluate their structural stability. The DHQD─ZINC14981770 complex reached equilibrium with an average RMSD of 2.3416 ± 0.0069 Å, while the DHQD─CA complex exhibited a slightly lower RMSD of 2.3142 ± 0.00854 Å. Although the difference between these two values was not statistically significant (P > 0.05), the slightly higher RMSD of DHQD─ZINC14981770 suggests that this complex underwent minor conformational adjustments. A block analysis over 10 ns intervals was conducted to confirm the statistical significance of these results (Table 4). However, these fluctuations did not affect its overall stability, as indicated by the consistent RMSD values throughout the simulation. Interactions between the ligand and the flexible loop regions of DHQD may have contributed to maintaining the stability of this complex (Fig. 3).

**Table 4:** Block Analysis of RMSD, RMSF, and Rg for DHQD-Ligand Complexes

Complex	RMSD (Å) (Mean ± SD)	RMSF (Å) (Mean ± SD)	Rg (Å) (Mean ± SD)	P-value (RMSD)	P-value (RMSF)	P-value (Rg)
DHQD─ZINC14981770	2.3416 ± 0.0069	7.85 ± 3.5	14.6693 ± 0.0019	P > 0.05	P > 0.05	P > 0.05
DHQD─ZINC14741224	1.5792 ± 0.0052	7.30 ± 3.1	14.6298 ± 0.0018	P > 0.05	P > 0.05	P > 0.05
DHQD─ZINC14743698	1.9306 ± 0.0043	8.15 ± 4.2	14.6762 ± 0.0021	P > 0.05	P > 0.05	P > 0.05
DHQD─ZINC13165465	2.0799 ± 0.0132	7.90 ± 3.6	14.8134 ± 0.0022	P > 0.05	P > 0.05	P > 0.05
DHQD─ZINC8442077	1.8743 ± 0.00774	8.05 ± 4.3	14.7576 ± 0.0022	P > 0.05	P > 0.05	P > 0.05
DHQD─CA	2.3142 ± 0.00854	8.35 ± 4.5	14.8969 ± 0.0036	-	-	-

Similarly, the DHQD─ZINC14741224 complex demonstrated a stable trajectory, achieving equilibrium after 7 ns and maintaining an average RMSD of 1.5792 ± 0.0052 Å, lower than DHQD─CA. The lower RMSD value indicates a highly stable interaction, likely due to strong hydrogen bonding and hydrophobic interactions with residues in the flexible loop regions of DHQD. The reduced flexibility observed in these complexes further highlights its stability, consistent with the docking results, where ZINC14741224 demonstrated favourable binding affinity (Fig. 3).

The DHQD─ZINC14743698 and DHQD─ZINC13165465 complexes exhibited average RMSD values of 1.9306 ± 0.0043 Å and 2.0799 ± 0.0132 Å, respectively, both of which were lower than the reference DHQD─CA complex. These values suggest these complexes maintained stable conformations during the simulation, reflecting ligand–protein strong interactions. Block analysis over 10 ns intervals further confirmed the consistency of these RMSD values (Table 4). The reduced flexibility in these complexes could be attributed to their ability to form stable interactions with critical active site residues, as observed during the docking analysis (Fig. 3). The DHQD─ZINC8442077 complex reached equilibrium after 3 ns, with an average RMSD of 1.8743 ± 0.00774 Å, which was also lower than DHQD─CA. Despite initial fluctuations, the complex stabilized, indicating a rigid binding mode within the active site. Overall, the RMSD analysis of all five ligand-DHQD complexes revealed that they formed stable and rigid systems during the 50 ns MD simulation, aligning with their docking energies and pharmacokinetic properties (Fig. 3).

**Fig. 4:**
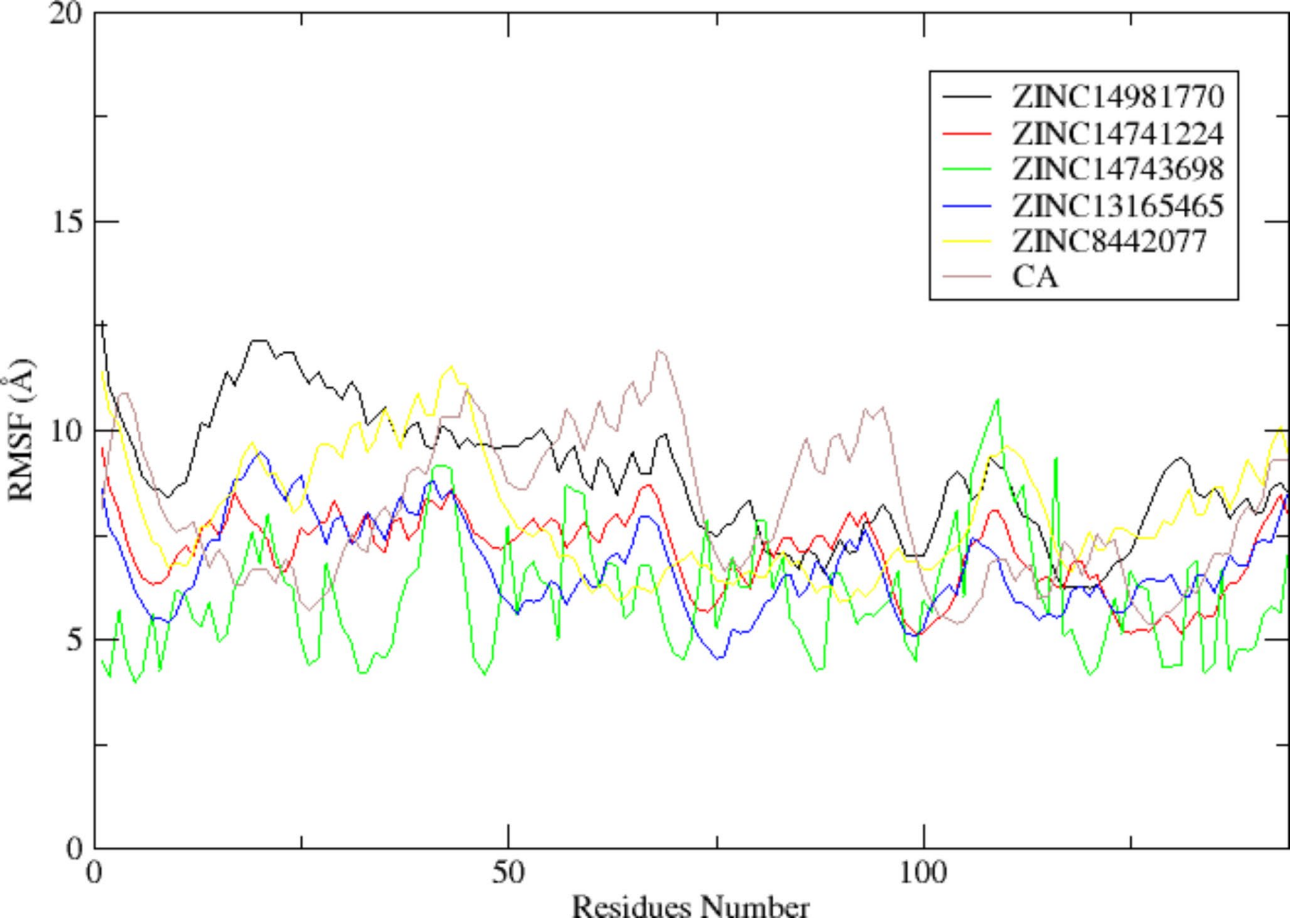
illustrates the results of a 50 ns MD simulation (RMSF analysis) conducted on the DHQD─ZINC14981770, DHQD─ZINC14741224, DHQD─ZINC14743698, DHQD─ZINC13165465, DHQD─ZINC8442077, and DHQD─CA complexes

**Fig. 5:**
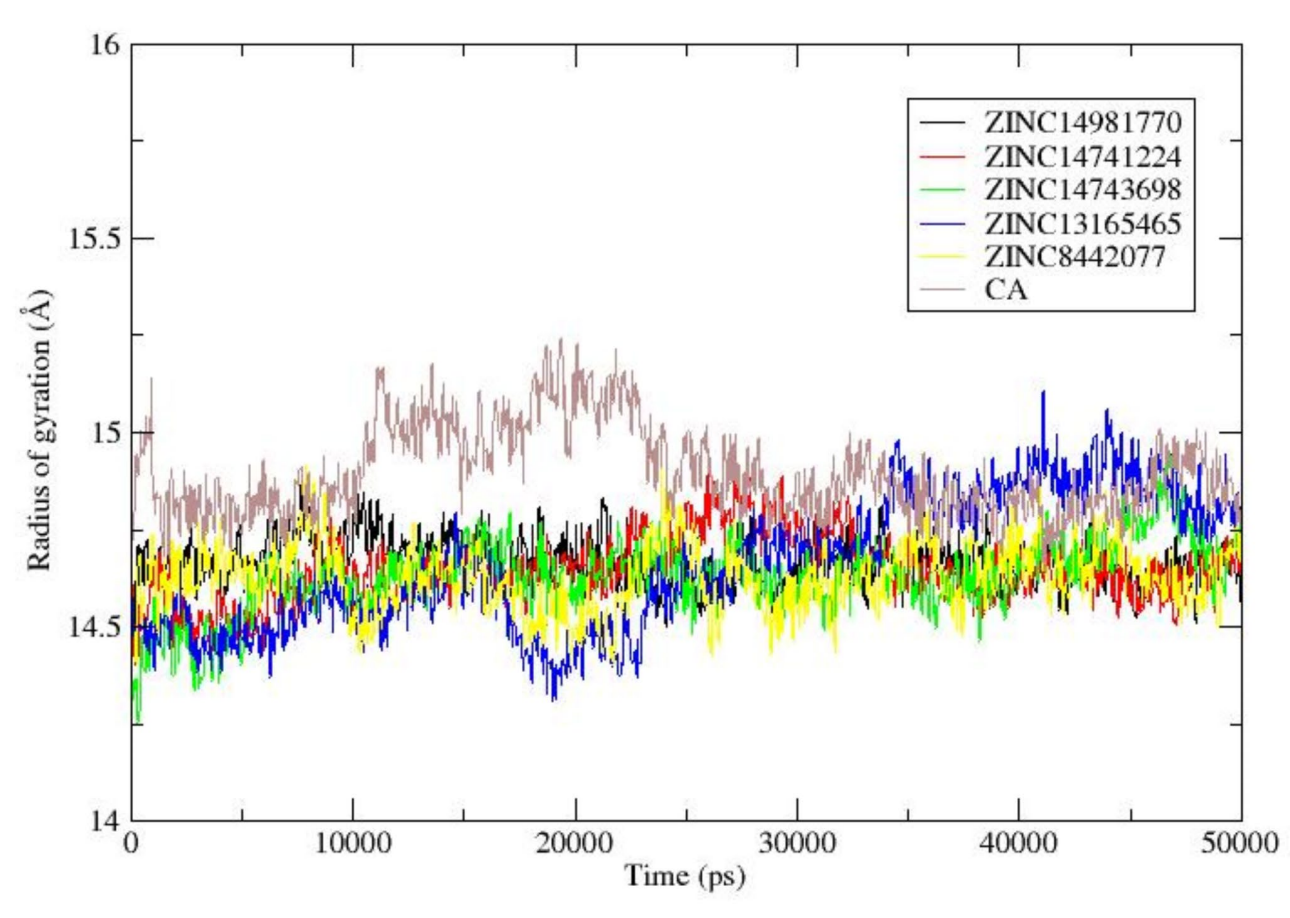
presents the outcomes of a 50 ns MD simulation (Radius of gyration analysis) performed on the DHQD─ZINC14981770, DHQD─ZINC14741224, DHQD─ZINC14743698, DHQD─ZINC13165465, DHQD─ZINC8442077, and DHQD─CA complexes

**Fig. 6:**
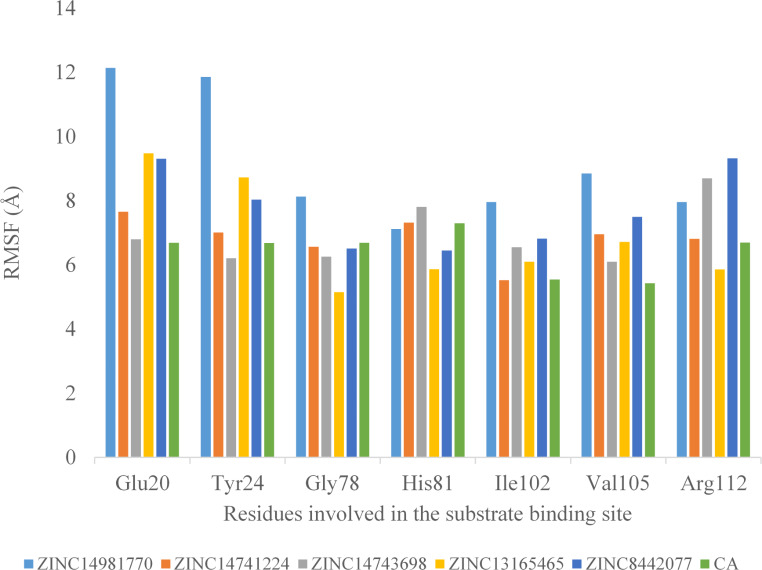
Illustrates the variation in DHQD residues within the substrate binding site following a 50ns MD simulation.

RMSF was used to investigate the flexibility of individual amino acid residues in the DHQD-ligand complexes, providing an understanding of the regions most affected by ligand binding (Fig. 4). In the DHQD─ZINC14981770 complex, most residues exhibited low RMSF values (< 10 Å), except for a few regions such as the N-terminal (Met1─Glu3), ω-loop (Asn13─Leu36), and C-terminal (Thr147), which had higher fluctuations (> 10 Å). These high RMSF values could indicate regions of greater flexibility that did not form stable interactions with the ligand. The DHQD─ZINC14741224, DHQD─ZINC14743698, and DHQD─ZINC13165465 complexes displayed similar trends, with most residues showing low RMSF values, particularly in the α-helix and β-sheet regions. A block analysis of RMSF was performed, and it was found that fluctuations in flexible loop regions did not significantly impact the overall stability of the complexes. The interactions between these ligands and residues in the flexible loop regions contributed to the reduced fluctuations observed in these complexes. This improved rigidity is consistent with the low RMSD values and docking results, which indicated ligand–protein solid interactions (Fig. 5). The DHQD─ZINC8442077 complex also demonstrated minimal fluctuations, except for residues in the ω-loop and C-terminal regions. In comparison, the DHQD─CA complex exhibited slightly higher RMSF values, suggesting that the selected ligands induced greater rigidity in the protein structure, contributing to the overall stability of the complexes (Figs. 4 and 6).

The radius of gyration (Rg) was employed to evaluate the compactness of the DHQD-ligand complexes, indicating whether the protein structures remained folded or underwent unfolding during the MD simulation. The DHQD─ZINC14981770 complex exhibited a mean Rg of 14.6693 ± 0.00195 Å, which was comparable to the DHQD─CA complex (14.8969 ± 0.00364 Å). The Rg values observed in both complexes indicate that they maintained a stable, folded structure throughout the simulation (Fig. 5). Similarly, the DHQD─ZINC14741224, DHQD─ZINC14743698, DHQD─ZINC13165465, and DHQD─ZINC8442077 complexes demonstrated stable Rg values, ranging from 14.62 to 14.95 Å. These values suggest that the ligands did not induce significant structural changes, and the protein–ligand complexes retained their compactness. The preservation of the folded state aligns with the low RMSD and RMSF values, further confirming the stability of these complexes (Fig. 5).

The MD simulation results confirm the docking predictions, where all five selected ligands exhibited strong binding affinities and stable interactions with DHQD. The stability of these complexes, as indicated by their low RMSD, minimal residue fluctuations (RMSF), and consistent Rg values, suggests that these ligands can form stable interactions with the protein's active site. A block analysis further confirmed that the RMSD, RMSF, and Rg values were not statistically significant over the 50 ns simulation period, which ensures the strength of these results (Table 4). The correlation between the docking scores and MD simulation outcomes provides confidence in the potential of these compounds as viable inhibitors of DHQD. These findings imply that the selected ligands could serve as lead compounds for further optimization and in vitro validation, particularly in drug discovery targeting DHQD.

The MD simulation results suggest that five selected compounds show strong potential as inhibitors for DHQD, a key enzyme related to tuberculosis. Each compound showed stable, strong binding to the enzyme’s active site, meaning they fit well within the structure and could effectively disrupt DHQD’s function. Stability indicators, such as low RMSD values, which showed that the compounds maintained better interactions with DHQD without causing the improper folding of the enzyme. These findings support the earlier predictions from docking studies and support moving forward with these compounds for further research (Fig. 6).

### Free binding energy calculation using MMGBSA

The Molecular Mechanics Generalized Born Surface Area (MM-GBSA) method was employed following a 50 ns molecular dynamics (MD) simulation to evaluate the free binding energy of the ligand–protein complexes. MM-GBSA integrates multiple energy components such as electrostatic interactions, van der Waals forces, nonpolar and polar solvation energies, and the unbound system's free energy to comprehensively assess the interaction strength between a ligand and its target protein (Xu et al. 2015). This method provides a more rigorous and accurate estimate of the free binding energy than the preliminary docking scores, which serve as an initial screening tool. Free binding energy is a vital parameter in drug discovery, as it provides an understanding of the stability and affinity of ligand–protein complexes, helping predict a compound's potential as a drug candidate. The results of the MM-GBSA analysis revealed that ZINC14981770 exhibited the lowest free binding energy, calculated at -32.70 ± 0.1596 kcal/mol, indicating the most vital interaction with the DHQD protein among all the ligands tested. This suggests that ZINC14981770 is energetically more favourable and likely to form a stable complex with DHQD than the other ligands. Following this, ZINC14741224 and ZINC14743698 also displayed low binding energies of -29.67 ± 0.2316 kcal/mol and -28.79 ± 0.3186 kcal/mol, respectively, further demonstrating their potential for stable interactions with DHQD. ZINC13165465 (-24.04 ± 0.1856 kcal/mol) and ZINC8442077 (-19.79 ± 0.1362 kcal/mol) had slightly higher binding energies but still performed better than the reference ligand CA, which exhibited a free binding energy of -10.62 ± 0.1630 kcal/mol. These MMGBSA results provide a more accurate reflection of the binding potential compared to the initial docking scores, highlighting ZINC14981770 as the most promising candidate. The significantly higher binding energy of CA indicates weaker interactions with DHQD, implying that it is less stable than the newly evaluated ligands (Table 5).

**Table 5:** Free binding energy obtained using MM-GBSA analysis

	**ZINC14981770**	**ZINC14741224**	**ZINC14743698**	**ZINC13165465**	**ZINC8442077**	**CA**
$\Delta$G_vdw_	─42.77 ± 0.1315	─42.27 ± 0.2019	─53.21 ± 0.2533	─33.31 ± 0.1875	─34.39 ± 0.1369	─14.25 ± 0.2623
$\Delta$G_ele_	─160.18 ± 0.9198	─100.79 ± 0.7253	─87.24 ± 1.1554	─3.79 ± 0.2106	─9.71 ± 0.1927	─100.46 ± 1.1754
$\Delta$G_polar_	174.31 ± 0.8435	117.42 ± 0.7606	116.42 ± 0.9087	16.37 ± 0.1517	27.57 ± 0.2212	105.96 ± 1.1975
$\Delta$G_nonpolar_	─4.06 ± 0.0056	─4.01 ± 0.0177	─4.76 ± 0.0078	─3.31 ± 0.0245	─3.26 ± 0.0084	─1.87 ± 0.0300
$\Delta$G_gas_	─202.95 ± 0.9434	─143.07 ± 0.7602	─140.45 ± 1.1131	─37.10 ± 0.2165	─44.10 ± 0.2233	─114.72 ± 1.2058
$\Delta$G_solv_	170.25 ± 0.8451	113.40 ± 0.7568	111.66 ± 0.9100	13.06 ± 0.1520	24.30 ± 0.2181	104.10 ± 1.1946
$\Delta$G_MM-GBSA_	─32.70 ± 0.1596	─29.67 ± 0.2316	─28.79 ± 0.3186	─24.04 ± 0.1856	─19.79 ± 0.1362	─10.62 ± 0.1630

The MM-GBSA free binding energy results closely align with the findings from molecular docking and MD simulations. In the docking analysis, ZINC14981770 had the lowest binding energy of -8.99 kcal/mol, corroborating the MM-GBSA result that highlighted this ligand as having the most favourable interaction with DHQD. Similarly, ZINC14741224 and ZINC14743698, which had binding energies of -8.98 kcal/mol and -8.87 kcal/mol, respectively, in the docking analysis, exhibited comparably low MM-GBSA free binding energies, further reinforcing the strong affinity of these ligands for DHQD.

Moreover, the MD simulation results, particularly the Root Mean Square Deviation (RMSD) and Root Mean Square Fluctuation (RMSF) analyses indicated that these ligands formed stable complexes with minimal fluctuations over the 50 ns simulation period. The RMSD values for ZINC14981770 and ZINC14741224 were notably low, reflecting stable protein–ligand interactions maintained throughout the simulation. These results are consistent with the MM-GBSA analysis, where these ligands exhibited the most favourable free binding energies, highlighting their firm interaction profiles. The consistency between docking, MD simulation, and MM-GBSA analyses supports these methods' reliability in predicting the ligands' stability and binding affinity. The correlation between docking, MD simulation, and MM-GBSA analyses suggests that ZINC14981770, ZINC14741224, and ZINC14743698 are promising candidates for further investigation, potentially advancing to in vitro validation studies. As demonstrated across multiple computational techniques, the stability and strong binding affinity of these ligands position them as lead compounds for drug development targeting DHQD. A detailed breakdown of the energy components contributing to the free binding energy revealed that van der Waals interactions were dominant in stabilizing the complexes, particularly for ZINC14981770, which exhibited the most favourable van der Waals energy. This is consistent with the hydrophobic nature of many vital residues in the DHQD binding pocket, suggesting that ligands with optimized hydrophobic interactions can further improve binding stability. Electrostatic interactions also contributed significantly, particularly for ZINC14741224, which demonstrated electrostatic solid energy contributions, likely due to favourable hydrogen bonding with polar residues within the DHQD active site. These findings imply that future ligand optimization efforts could focus on improving both hydrophobic and electrostatic interactions to increase binding affinity. The nonpolar solvation energy was relatively low across all complexes. In contrast, the polar solvation energy, which typically opposes binding due to desolvation costs, was higher but manageable, particularly for the top-performing ligands. The ability of ZINC14981770 and other lead compounds to maintain favourable van der Waals and electrostatic contributions while minimizing the destabilizing effects of solvation indicates their suitability as drug candidates.

The MM-GBSA analysis supports the potential of ZINC14981770, ZINC14741224, and ZINC14743698 as lead compounds for DHQD inhibition. Their low free binding energies and consistent findings from docking and MD simulations suggest that these compounds can form stable, high-affinity complexes with DHQD, which is critical for effective drug action. Given the correlation between computational binding energy predictions and experimental data reported in the literature, these compounds warrant further investigation through in vitro and in vivo studies. The weak performance of the reference ligand CA, both in terms of free binding energy and MD simulation stability, emphasizes the advantage of computational screening in identifying higher alternatives. The newly identified ligands exhibit better stability and interaction potential than CA, suggesting they could offer improved efficacy in inhibiting DHQD's activity. The combination of docking, MD simulations, and MMGBSA provides a robust framework for evaluating the binding affinity and stability of potential drug candidates, reinforcing the reliability of our computational screening approach.

## Conclusion

In this study, we identified 9,699 compounds through virtual screening, and after filtering based on Lipinski’s Rule of Five and docking analysis, eight lead compounds with strong binding affinities were selected. Five compounds—ZINC14981770, ZINC14741224, ZINC14743698, ZINC13165465, and ZINC8442077—demonstrated favourable pharmacokinetics and toxicity profiles and were chosen for further evaluation. MD simulations and MM-GBSA analysis showed these compounds formed stable complexes with DHQD, with binding energies significantly lower than the reference ligand, CA. Therefore, further in vitro or in vivo testing is necessary to confirm their efficacy as DHQD inhibitors. Future studies using more advanced computational methods, such as QM/MM calculations, which will provide deeper understanding of the binding mechanisms.

## Data Availability

No datasets were generated or analysed during the current study.

## References

[CR1] Berman HM, Westbrook J, Feng Z, Gilliland G, Bhat TN, Weissig H, and Bourne PE (2000), The Protein Data Bank Nucleic Acids Research, 28, 235–242. *URL: *www.rcsb.orgCitation. 10.1093/nar/28.1.235 PMC102472 10592235

[CR2] Case D.A., Aktulga H.M., K. Belfon, I.Y. Ben-Shalom, J.T. Berryman, S.R. Brozell, D.S. Cerutti, T.E. Cheatham, III, G.A. Cisneros, V.W.D. Cruzeiro, T.A. Darden, N. Forouzesh, G. Giambaşu, T. Giese, M.K. Gilson, H. Gohlke, A.W. Goetz, J. Harris, S. Izadi, S.A. Izmailov, K. Kasavajhala, M.C. Kaymak, E. King, A. Kovalenko, T. Kurtzman, T.S. Lee, P. Li, C. Lin, J. Liu, T. Luchko, R. Luo, M. Machado, V. Man, M. Manathunga, K.M. Merz, Y. Miao, O. Mikhailovskii, G. Monard, H. Nguyen, K.A. O’Hearn, A. Onufriev, F. Pan, S. Pantano, R. Qi, A. Rahnamoun, D.R. Roe, A. Roitberg, C. Sagui, S. Schott-Verdugo, A. Shajan, J. Shen, C.L. Simmerling, N.R. Skrynnikov, J. Smith, J. Swails, R.C. Walker, J. Wang, J. Wang, H. Wei, X. Wu, Y. Wu, Y. Xiong, Y. Xue, D.M. York, S. Zhao, Q. Zhu, and P.A. Kollman (2023), Amber 2023, University of California, San Francisco.

[CR3] Chaudhuri S, Duncan K, Graham LD, Coggins JR (1991) Identification of the active-site lysine residues of two biosynthetic 3-dehydroquinases. Biochem J 275(1):1–6 1826831 10.1042/bj2750001 PMC1150004

[CR4] Cheng F, Li W, Zhou Y, Shen J, Wu Z, Liu G, Lee PW, and Tang Y (2012), admetSAR: a comprehensive source and free tool for assessment of chemical ADMET properties. 3099–3105. 10.1021/ci300367a 23092397

[CR5] Genheden S, Ryde U (2015) The MM/PBSA and MM/GBSA methods to estimate ligand-binding affinities. Expert Opin Drug Discov 10(5):449–461 25835573 10.1517/17460441.2015.1032936 PMC4487606

[CR6] Ghasemi F, Zomorodipour A, Karkhane AA, Khorramizadeh MR (2016) In silico designing of hyper-glycosylated analogs for the human coagulation factor IX. J Mol Graph Model 68:39–47 27356208 10.1016/j.jmgm.2016.05.011

[CR7] Gourley DG, Shrive AK, Polikarpov I, Krell T, Coggins JR, Hawkins AR, Sawyer L (1999) The two types of 3-dehydroquinase have distinct structures but catalyze the same overall reaction. Nat Struct Mol Biol 6(6):521–525 10.1038/9287 10360352

[CR8] Harris J, Kleanthous C, Coggins JR, Hawkins AR, Abell C (1993) Different mechanistic and stereochemical courses for the reactions catalyzed by type I and type II dehydroquinases. J Chem Soc, Chem Commun 13:1080–1081

[CR9] He G, Qiu M, Li R, Ouyang L, Wu F, Song X, Li C, Xiang M, Yu L (2012) Multicomplex-based pharmacophore-guided 3D-QSAR studies of N-substituted 2′-(aminoaryl)benzothiazoles as aurora-A inhibitors. Chem Biol Drug des. 10.1111/j.1747-0285.2012.01366.x 22381081 10.1111/j.1747-0285.2012.01366.x

[CR10] Herrmann KM (1995) The shikimate pathway: early steps in the biosynthesis of aromatic compounds. Plant Cell 7(7):907 12242393 10.1105/tpc.7.7.907 PMC160886

[CR11] Holderbach S, Adam L, Jayaram B, Wade RC, Mukherjee G (2020) RASPD+: fast protein-ligand binding free energy prediction using simplified physicochemical features. Front Mol Biosci 7:601065 33392260 10.3389/fmolb.2020.601065 PMC7773945

[CR12] Isa MA, Majumdhar RS, Haider S, Kandasamy S (2018) Molecular modelling and dynamic simulation of UDP-N-acetylglucosamine 1-carboxyvinyltransferase (MurA) from Mycobacterium tuberculosis using in silico approach. Inform Med Unlocked 12:56–66. 10.1016/j.imu.2018.06.007

[CR13] Johansson MU, Zoete V, Michielin O, Guex N (2012) Defining and searching for structural motifs using DeepView/Swiss-PdbViewer. BMC Bioinform 13(1):173 10.1186/1471-2105-13-173 PMC3436773 22823337

[CR14] Jothieswari D, Bhaskar Reddy K (2015) Molecular Docking studies of potential chemical inhibitors on multi-drug resistance genes in MTB. Int J Innov Drug Discov 5(1):40–45

[CR15] Kumari R, Dalal V (2022) Identification of potential inhibitors for LLM of Staphylococcus aureus: structure-based pharmacophore modeling, molecular dynamics, and binding free energy studies. J Biomol Struct Dyn 40(20):9833–9847 34096457 10.1080/07391102.2021.1936179

[CR16] Kumari R, Rathi R, Pathak SR, Dalal V (2022) Structural-based virtual screening and identification of novel potent antimicrobial compounds against YsxC of Staphylococcus aureus. J Mol Struct 1255:132476

[CR17] Lipinski CA, Lombardo F, Dominy BW, Feeney PJ (1997) Experimental and computational approaches to estimate solubility and permeability in drug discovery and development settings.". Adv Drug Deliv Rev 23(1–3):3–25 10.1016/s0169-409x(00)00129-0 11259830

[CR18] Morris GM, Goodsell DS, Halliday RS, Huey R, Hart WE, Belew RK, Olson AJ (1998) Automated docking using a Lamarckian genetic algorithm and an empirical binding free energy function. J Comput Chem 19(14):1639–1662

[CR19] Pettersen EF, Goddard TD, Huang CC, Couch GS, Greenblatt DM, Meng EC, Ferrin TE (2004) UCSF Chimera-a visualization system for exploratory research and analysis. J Comput Chem 25(13):1605–1612 15264254 10.1002/jcc.20084

[CR20] Pires DE, Blundell TL, Ascher DB (2015) pkCSM: predicting small-molecule pharmacokinetic and toxicity properties using graph-based signatures. J Med Chem 58(9):4066–4072 25860834 10.1021/acs.jmedchem.5b00104 PMC4434528

[CR21] Ramachandran S, Kota P, Ding F, Dokholyan NV (2011) Automated minimization of steric clashes in protein structures. Prot Struct Funct Bioinform 79(1):261–270 10.1002/prot.22879 PMC3058769 21058396

[CR22] Rowland K (2012) Totally drug-resistant TB emerges in India. Nature 1:9797

[CR23] Sander T, Joel F, von Korff M, Christian R (2015) DataWarrior: an open-source program for chemistry aware data visualization and analysis. J Chem Inf Model 55(2):460–473 25558886 10.1021/ci500588j

[CR24] Suvaithenamudhan S, Parthasarathy S (2017) Molecular dynamics simulations of novel potential inhibitors for penicillin binding protein 2B of the resistant 5204 strain of Streptococcus pneumoniae. Curr Comput Aided Drug des 13(3):234–248 28260518 10.2174/1573409913666170301120421

[CR25] Suvaithenamudhan S, Parthasarathy S (2023) In silico studies on potential inhibitors of the penicillin binding protein 2B (PBP2B) of the resistant G54 and intermediate-resistant hungary 19A–6 and SP195 strains of streptococcus pneumoniae. Lett Drug des Discov 20(7):863–880

[CR26] Veber DF, Johnson SR, Cheng H-Y, Smith BR, Ward KW, Kopple KD (2002) Molecular properties that influence the oral bioavailability of drug candidates. J Med Chem 45(12):2615–2623 12036371 10.1021/jm020017n

[CR27] World Health Organization (2023). Tuberculosis fact sheet. https://www.who.int/health-topics/tuberculosis#tab=tab

[CR28] Xu J, Yuan H, Ran T, Zhang Y, Liu H, Lu S, Xiong X, Xu A, Jiang Y, LuChen TY (2015) A selectivity study of sodium-dependent glucose 567 cotransporter 2/sodium dependent glucose cotransporter 1 inhibitors 568 by molecular modeling. J Mol Recognit 28(8):467–479 25753971 10.1002/jmr.2464

[CR29] Zhang X, Zhang S, Hao F, Lai X, Yu H, Huang Y, Wang H (2005) Expression, purification and properties of shikimate dehydrogenase from MTB. J Biochem Mol Biol 38(5):624 16202245 10.5483/bmbrep.2005.38.5.624

